# Editorial and Miscellaneous

**Published:** 1862-02

**Authors:** 


### EDITORIAL.

*Apologetic.*—The unusual pressure of other engagements must be our apology for the brevity of our editorial columns the present month. We have several valuable original communications on hand which were received too late for insertion in our present number. The important subjects treated in our selections will, we are confident, be fully appreciated by readers.

*A Hint.*—A contemporary complains that some of his neighbors have been making "cowardly insinuations." It is very much in the condition of the fellow who did not attend the party "because of a *hint* he received. "How was that?" inquired a sympathizing friend." "Because when I went to the door of the house, the master ordered the servants to kick me out—I accepted the *insinuation* and left."

*Death Bed Repentance.*—The gentlemen who seceded from Rush Medical College two or three years ago, expecting thereby to bring it to destruction, have become lachrymose, and, in the last number of their organ, adopt as their own the language of the almanac and exclaim: "The man who tries to build himself up by pulling others down, is like one who sits himself in a wheelbarrow, and tries to wheel himself to glory."

We endorse the idea, and suggest that it cannot be done, even though Mr. Sylvester Lind takes hold of the handles of the barrow.

*Anonymous.*—Some one, with a feminine chirography, sends us an advertising puff of Homœopathy, having the recent infinitesimal army appointments as a text, requesting us to insert it, "with comments." The advertisement is declined. We advertise to a limited extent to accommodate dealers in professional wares, and to aid in the support of our periodical; but we insert no advertisements free, neither do we accept for any pay the advertisements of Lock Hospitals, Patent pills, Abortionists, Spiritualists, Hydropathists or Homœopathists, or anything of that ilk.

Homœopathists have been appointed in the army, but we are gratified in being able to state that thus far they have not been appointed as homœopathists. On their examination and their credentials, they have been exceedingly careful to hide their homœopathy. When asked how they would treat diseases, they have been very careful to answer in accordance with the principles of scientific medicine, and not in accordance with homœopathic teachings. In brief, their *status* in the grand army is precisely of the character of that of secessionists and other traitors who cloak their infamous principles in the garb of loyalty and patriotism.

If Homœopathy can glorify over this position of those of its disciples who have crept into the medical service of the Government, we congratulate them on their proficiency in the science of swindling and fraud, and cheerfully express the hope that it will speedily secure the time-honored reward of similar proficiency.

The chirography of our correspondent is suggestive of hysteria, and we cease to wonder at her belief. Or is it the production of a neuter? Reflection leaves us in a quandary, and we are obliged to leave the whole matter in Small hands.

---

*Notes on the Surgery of the War in the Crimea, with Remarks on the Treatment of Gunshot Wounds. By George H. B. Macleod, M. D., F. R. C. S., &c. J. B. Lippincott & Co.; Philadelphia:* 1862. *Pp.* 402.

This is a valuable and interesting treatise, and although perhaps especially intended for our military surgeons, nevertheless is worthy reading and a place in every physician's library. It is timely, written with great good sense, and shows a familiarity with practical details every day demanded of the military surgeon, which must commend it to general favor.

---

*Want of space* prevents the notice of the treatise on the Disease of the Joints, the reception of which was acknowledged last month. It will keep, however, and meanwhile we advise its purchase and perusal by all interested in the subject.

